# In silico and in vitro analysis of the impact of single substitutions within EXO-motifs on Hsa-MiR-1246 intercellular transfer in breast cancer cell

**DOI:** 10.1007/s13353-022-00730-y

**Published:** 2022-11-17

**Authors:** Agnieszka Rybarczyk, Tomasz Lehmann, Ewa Iwańczyk-Skalska, Wojciech Juzwa, Andrzej Pławski, Kamil Kopciuch, Jacek Blazewicz, Paweł P. Jagodziński

**Affiliations:** 1 Institute of Computing Science, Poznan University of Technology, Piotrowo 2, 60-965 Poznan, Poland; 2 Department of Biochemistry and Molecular Biology, Poznan University of Medical Sciences, Fredry 10, 61-701 Poznan, Poland; 3 Biotechnology and Food Microbiology, Poznan University of Life Sciences, Wojska Polskiego 48, 60-627 Poznan, Poland; 4 Institute of Human Genetics, Polish Academy of Sciences, Strzeszyńska 32, 60-479 Poznan, Poland; 5 Institute of Bioorganic Chemistry, Polish Academy of Sciences, Noskowskiego 12/14, 61-704 Poznan, Poland

**Keywords:** Breast cancer, EXO-motifs, Extracellular vesicles, MicroRNA, RNA structure prediction

## Abstract

MiR-1246 has recently gained much attention and many studies have shown its oncogenic role in colorectal, breast, lung, and ovarian cancers. However, miR-1246 processing, stability, and mechanisms directing miR-1246 into neighbor cells remain still unclear. In this study, we aimed to determine the role of single-nucleotide substitutions within short exosome sorting motifs — so-called EXO-motifs: GGAG and GCAG present in miR-1246 sequence on its intracellular stability and extracellular transfer. We applied in silico methods such as 2D and 3D structure analysis and modeling of protein interactions. We also performed in vitro validation through the transfection of fluorescently labeled miRNA to MDA-MB-231 cells, which we analyzed by flow cytometry and fluorescent microscopy. Our results suggest that nucleotides alterations that disturbed miR-1246 EXO-motifs were able to modulate miRNA-1246 stability and its transfer level to the neighboring cells, suggesting that the molecular mechanism of RNA stability and intercellular transfer can be closely related.

## Introduction

MicroRNAs (miRNAs) are a class of small non-coding RNAs that play a crucial role in a broad variety of physiological processes (Krol et al. 2010). They decrease mRNA levels through post-transcriptional degradation and translational repression. miRNA genes are transcribed into primary miRNAs (pri-miRNAs) and processed by Drosha to form precursor miRNAs (pre-miRNAs). Pre-miRNAs are exported into the cytoplasm by the exportin complex. Next, the pre-miRNAs undergo processing by Dicer complexes to become finally mature miRNAs (Bartel 2018; O’Brien et al. 2018).

Since deregulation of miRNAs is associated with many pathological conditions, including inflammation, cancer, cardiovascular diseases, viral diseases, and neurological disorders, miRNA has emerged as promising therapeutic target (Ha 2011; Walayat et al. 2018; Condrat et al. 2020; Wang et al. 2020). Therefore, an approach using miRNA mimics has been developed. Synthetic, double-stranded, short RNAs (dsRNAs) have been designed to reconstitute and simulate natural endogenous miRNAs (Zhang et al. 2013; Nogimori et al. 2019). Such synthetic RNA duplexes of 21–24 nucleotides (nts) in length are introduced to mammalian cells, where they initiate the RNA interference (RNAi) process by which dsRNA causes highly specific annealing and degradation of target mRNAs (Liang et al. 2013).

Transfected synthetic pre-miRNA is processed into siRNAs (small interfering RNAs) using endogenous proteins including Dicer. The double-stranded siRNAs are then directly incorporated into the RNA-induced silencing complex (RISC) containing Ago2 (Argonaute RISC Catalytic Component 2) protein (Liang et al. 2013). One of two strands of the siRNA duplex is released from the complex, leaving only the guide strand associated with Ago2 (Liang et al. 2013). The guide strand siRNA directs the RISC to substrate mRNA, which is cleaved by Ago2, resulting in sequence-specific mRNA cleavage through perfect base-pairing of siRNA with target mRNA (Liang et al. 2013). Additionally, various siRNAs may have different affinities for Ago2 protein (Liang et al. 2013). Moreover, chemical modifications often present in mimics improve their stability, facilitate guide miRNA loading to RISC, and selectively exclude the passenger strand (Jin et al. 2015).

Delivery of miRNA mimics into cells can bypass the endogenous miRNA biogenesis pathway. Therefore, transient transfection can efficiently deliver miRNA mimics into in vitro cultured mammalian cells and has been taken for granted as a fast, easy, and economical way to gain insight into the functions and mechanisms of gene expression (Jin et al. 2015). Transfection of siRNAs leads to altered expression of many miRNAs and genes and can cause changes in the protein level (Liang et al. 2013). The endogenous miRNAs and exogenous siRNAs can also be transferred intercellularly by the means of communication connections such as gap junctions, tunneling nanotubes, extracellular vesicles (EVs), free nucleic acids, or protein-associated RNAs (Lemcke et al. 2015; Charreau 2021; Lou 2020). MiRNA sorted to the secretory microvesicles (exosomes) and delivered to the recipient cells can exert special functional roles (Yue et al. 2020).

Exosomes are 40–100-nm nano-sized endosome-derived vesicles containing proteins, lipids, mRNA, and miRNAs released from many cell types into the extracellular space (Zhang et al. 2015). They can be selectively internalized by nearby or distant cells far from their releases, regulating recipient cells’ gene expression upon their content (Zhang et al. 2015). All eucaryotic cells secrete extracellular vesicles (EVs) in their normal and pathological circumstances. EVs can be broadly divided into two categories, ectosomes and exosomes. Ectosomes are vesicles that pinch off the surface of the plasma membrane via outward budding and include microvesicles, microparticles, and large vesicles in the size range of ~50 nm to 1 µm in diameter. Exosomes have a size ranging from ~40 to 160 nm (average ~100 nm) in diameter with an endosomal origin (Kalluri and LeBleu 2020; Pegtel and Gould 2019).

It has been shown that tumoral cells are a source of excessive amounts of EVs (extracellular vesicles) — released into the circulation and through their cargo to promote cancer progression and metastasis. Recent findings have highlighted miRNA molecules as key players in those processes (Hannafon et al. 2016; Torii et al. 2021). It has also been observed that miRNA species in exosomes and cells differed indicating some selection mechanism packing miRNAs into exosomes (Hannafon et al. 2016). Such an active sorting mechanism of exosomal miRNAs has been proposed in the recent literature (Zhang et al. 2015).

Generally, mature miRNAs are sorted into exosomes via four potential modes: (1) nSMase2-dependent pathway, (2) miRNA 3′-motif GGAG and sumoylated hnRNPs-dependent pathway; (3) 3′ miRNA sequence-dependent pathway where miRNAs that are preferentially sorted into exosomes contain more poly(U) than poly(A) at the 3′ end; (4) miRISC co-localized with the sites of exosome biogenesis (Zhang et al. 2015).

Computational analysis of miRNA sequences that are more abundant in exosomes than in cells led to the identification of specific short nucleotide motifs termed EXO-motifs (EVs export sequences) that may be involved in miRNAs selective transport into exosomes (Ragusa et al. 2017). While the full mechanism of sorting remains to be determined, it has been recognized that miRNA entering into the exosomes might be involved in interaction with different ribonucleoproteins, each controlling the exosomal loading of the specific set of miRNAs (Ragusa et al. 2017; Garcia-Martin et al. 2022). For instance, it has been demonstrated that EXO-motifs are recognized and bound by the heterogeneous ribonucleoprotein A2B1 (hnRNPA2B1) (Ragusa et al. 2017). Specifically, the sumoylated form of hnRNPA2B1 performs miRNA sorting into exosomes (Villarroya-Beltri et al. 2013). Therefore, the studies investigating EXO-motifs can lead to substantial improvement in EVs miRNA transfer to target cells, and what follows, causes the subsequent reduction in the expression of target genes, thereby offering the potential to enhance the effectiveness of miRNA delivery (Garcia-Martin et al. 2022; Martellucci et al. 2020). The study of EXO-motifs could shed some light also on other selective manners of intercellular miRNA shearing.

One of the exosomal miRNAs that has gained much attention recently is miR-1246. It has been reported to be highly expressed in blood plasma exosomes from patients affected by breast and colon cancers (Hannafon et al. 2016; Ogata-Kawata et al. 2014). It is known to be highly enriched in EVs derived from human cancer cells and to be more abundant in EVs derived from the breast cancer cell lines such as MCF7 and MDA-MB-231 (Hannafon et al. 2016). MiR-1246 originates from a small nuclear RNA RNU2-1 and is not generated in a classic Drosha- and Dicer-dependent pathway (Torii et al. 2021; Xu et al. 2019). It also contains, similarly to miR-122-5p (Li et al. 2021), two potential EXO-motifs: GGAG and GCAG, which overlap and share one nucleotide G (last position of GGAG and first of GCAG, namely 5′-GGAGCAG-3′). However, only one of the two abovementioned motifs, namely GCAG was studied with regard to miR-1246 transfer into exosomes (Xu et al. 2019).

Moreover, it has recently been shown that highly metastatic tumors induce drug resistance in endothelial cells by transporting miR-1246 through exosomes (Torii et al. 2021). The detailed mechanisms responsible for sorting miR-1246 into highly metastatic tumor-secreted EVs are still unclear and require further study (Torii et al. 2021); Dai et al. 2021). Defining the full miRNA sorting machinery involved in EVs secretion will improve the understanding of how circulating miRNAs change in disease, will help to better relate circulating miRNAs to their tissue of origin, and will open the possibility of regulating miRNA retention or secretion for therapeutic benefits (Garcia-Martin et al. 2022).

Since miR-1246 is known to have an essential impact on carcinogenic events in different tissues, studying the intercellular transfer of this miRNA can be of great importance in explaining the mechanisms of tumor-related migration and invasion, and finally, miR-1246 can also act as a therapeutic target (Bhagirath et al. 2018; Bakhsh et al. 2022).

To the best of our knowledge, EXO-motifs within miR-1246 have not been analyzed as an important factor regulating miR-1246 stability and intercellular migration. The GGAG EXO-motif is one of the over-represented motifs in exosomal miRNAs, including miR-1246 (Villarroya-Beltri et al. 2013; Wei et al. 2021), and was not investigated as an exportation signal for this miRNA. This is the reason why we decided to explore this motif in the present study.

The aim of this work was to check whether single substitutions within GGAG EXO-motif of miR-1246 could modulate its stability and intracellular transfer in vitro. Thus, we developed the two-step approach composed of computational analysis and biological experiment. First, we elaborated a computational workflow, dedicated to the multilevel analysis of different miR-1246 variants, where miRNAs were studied at the level of their primary (1D), secondary (2D), and tertiary (3D) structure. Next, miR-1246 sequence variants selected basing on the results of the aforementioned in silico analysis were subjected to the in vitro validation. Synthetic miRNAs were delivered to the breast cancer cells (MDA-MB-231) and analyzed qualitatively and quantitatively by flow cytometry and fluorescent microscopy to estimate the impact of EXO-motifs modifications on the miRNA transfer between cells.

## Material and methods

First, we conducted in silico analysis of all miRNA variants that differed from native miR-1246 by single substitution within EXO-motif GGAG. We studied the impact of a single nucleotide modification on the presence of potential consensus motifs associated with RNA binding proteins (RBPs), and we examined the secondary (2D) and tertiary (3D) structures of all EXO-motif GGAG variants in miR-1246. Based on the results of the in silico analysis, we selected modified miRNA sequences for the in vitro validation. Modified sequences within GGAG EXO-motif within miR-1246 were introduced as miRNA FITC labeled duplexes to MDA-MB-231 cells. The cells were then analyzed by flow cytometry and fluorescent microscopy. In this way, we measured the stability of miR-1246 and its variants in cells and their intercellular transfer.

### In silico data analysis

**Fig. 1:**
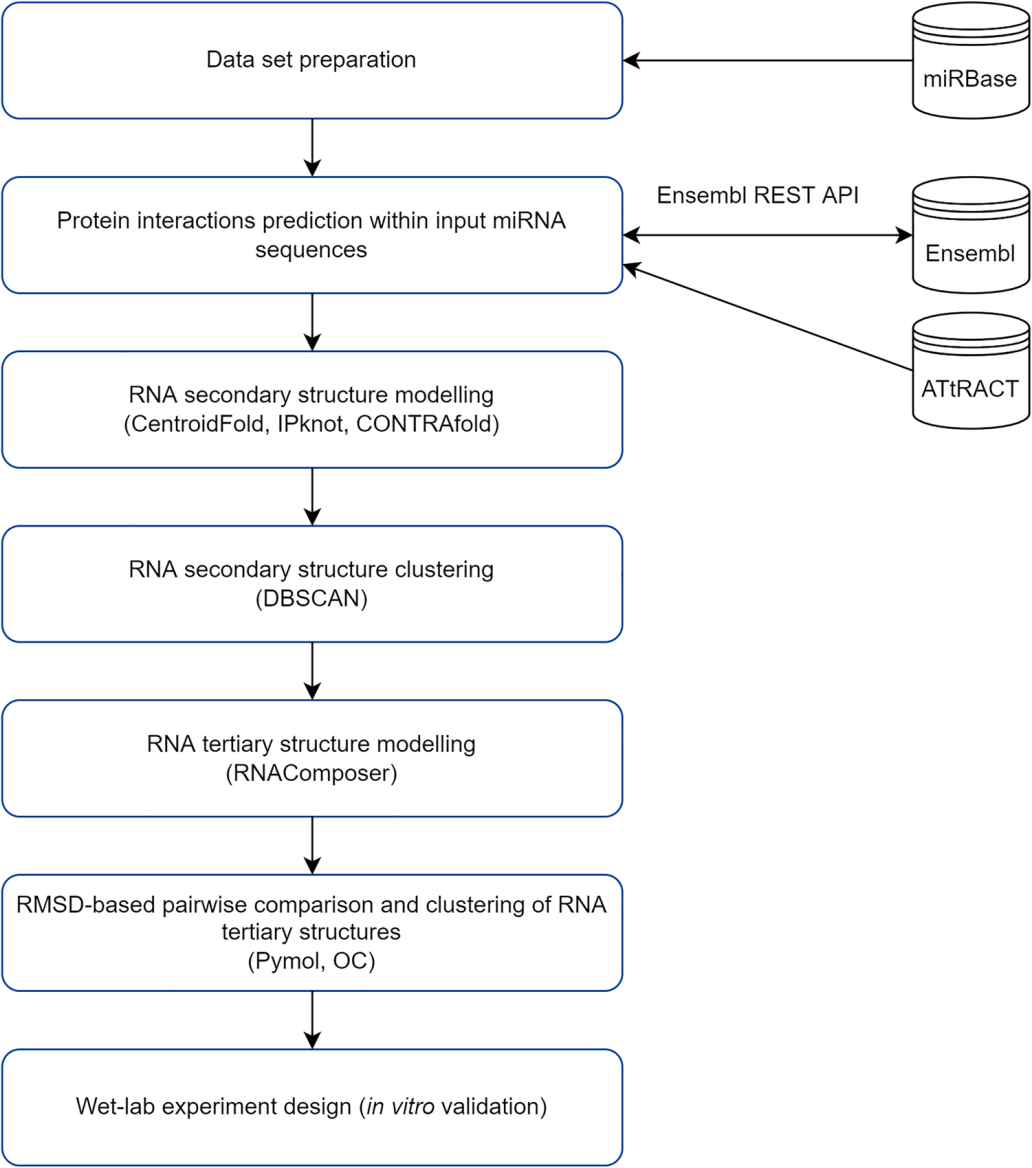
The workflow of the in silico study

The subsequent, fundamental steps of the in silico analysis are presented in Fig. 1. The workflow of the in silico study was designed and prepared to utilize the experience gained from the previously conducted research (Popenda et al. 2012; Szostak et al. 2014; Rybarczyk et al. 2015; Biesiada et al. 2016; Antczak et al. 2019).

#### Input miRNA sequence

The sequence of the hsa-miR-1246 (miR-1246) mimic (5$'$-AAUGGAUUUUUGGAGCAGG-3$'$) was retrieved from the miRBase database (http://www.mirbase.org, miRBase accession number equal to MIMAT0005898).

#### Data set preparation

According to Zhang et al. (2015) and Wei et al. (2021), short sequence motifs, termed EXO-motifs, can selectively control miRNA sorting into exosomes through interaction with RNA-binding proteins, such as hnRNPA2B1. Their directed mutagenesis or induced changes in hnRNPA2B1 expression levels can modulate the miRNA cargoes (Villarroya-Beltri et al. 2013). One of the over-represented motifs in EXOmiRNAs, including miR-1246, is EXO-motif GGAG (Villarroya-Beltri et al. 2013; Wei et al. 2021). In order to verify whether single nucleotide substitutions within the aforementioned motif can influence miR-1246 levels sorted into exosomes and transferred to the neighboring cells, we have generated 12 RNA (miRNA) sequences numbered from 1 to 12. They differed from native miR-1246 by single nucleotide within EXO-motif GGAG (c.f. Table 1).

**Table 1:**
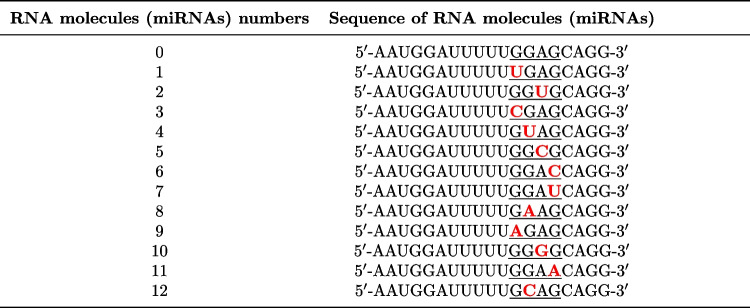
The sequences of RNA (miRNA) molecules used in the in silico data analysis

RNA number 0 refers to the native miR-1246 sequence. RNAs numbered from 1 to 12 differ from native miR-1246 by a single nucleotide within EXO-motif GGAG. The substituted nucleotides are denoted in red. Nucleotides forming EXO-motif are underlined

#### Protein interactions prediction

In order to find potential binding sites for possible protein interactions within analyzed miRNA sequences (c.f. Table 1), we developed our own approach and implemented it in a form of Python scripts. It requires the list of miRNA sequences in FASTA format, the database of consensus motifs, and the name of the organism of interest.

To provide the input database of consensus motifs, we downloaded the entire content of ATtRACT (Giudice et al. 2016). It is a database of experimentally validated RNA binding proteins (RBPs) and associative motifs, equipped with an efficient search engine to exploit the information about potential binding sites within RNA sequences provided as an input. It contains information about over 370 RBPs associated with 1583 consensus motifs.

In the first step, based on the aforementioned database, the input miRNA sequences were scanned for the appearance of RBPs motifs. Next, the sequences were grouped by the occurrence of motifs to avoid duplicates and to increase the efficiency of the algorithm. In the case of each motif, Ensemble gene ID of RBP was assigned to it and Ensembl REST API (Yates et al. 2015) was used to retrieve additional information such as Ensembl transcript ID, Ensembl protein ID, and list of Gene Ontology (GO) terms describing functional attributes of a given gene. For each gene associated with a motif, we used Ensembl REST API to retrieve its homologs. In the last step, the results obtained for the homologous genes were grouped together allowing us to investigate the miRNAs possessing binding sites for proteins having similar functions.

Additionally, we prepared Python scripts allowing us to calculate the number of the most commonly encountered motifs within the analyzed set of sequences and the miRNA ranking which presents input RNA sequences ordered by the number of potential binding sites for the homologous genes of RBPs.

#### RNA 2D structure modeling

RNA secondary structure of analyzed miRNA sequences (c.f. Table 1) was predicted using the following bioinformatics tools: CentroidFold (Sato et al. 2009), CONTRAFold (Do et al. 2006), and IPknot (Sato et al. 2011). CentroidFold predicts the RNA secondary structure improving its accuracy by means of generalized centroid estimators that maximize the expected weighted true predictions of base pairs in the predicted structure. CONTRAfold is a pseudoknot-free RNA secondary structure prediction method based on conditional log-linear models (CLLMs) that generalize on stochastic context-free grammars (SCFGs) by using discriminative training and feature-rich scoring. IPknot is a tool that requires the Vienna RNA Package as its base and is capable of predicting RNA secondary structures including a wide class of pseudoknots. It is implemented using integer programming. It is worth mentioning that IPknot can be considered as an extension of CentroidFold (Hamada et al. 2009).

#### RNA secondary structure clustering

Pairwise comparison of all considered secondary structures represented in dot-bracket notation was computed using Levenshtein distance (LD) (Levenshtein 1966) that measures the minimum number of edit operations (insertions, deletions, and substitutions) required to transform one string into the other. Next, based on the obtained comparison matrix, secondary structures were clustered using DBSCAN (density-based spatial clustering of applications with noise) (Ester et al. 1996). It is a well-known data clustering algorithm that is commonly used in data mining and machine learning.

#### RNA 3D structure modeling

RNAComposer (Popenda et al. 2012; Biesiada et al. 2016) was applied to predict RNA 3D structures for the input miRNA sequences. Computation started from running three RNAComposer-integrated algorithms for RNA secondary structure prediction: CentroidFold (Sato et al. 2009), CONTRAfold (Do et al. 2006), and IPknot (Sato et al. 2011). As an output, up to 10 RNA 3D models were obtained for each secondary structure topology.

RNAComposer is a user-friendly web-based system that allows for fully automated prediction of large RNA 3D models from canonical secondary structures. It uses the RNA FRABASE database (Popenda et al. 2008) serving as a dictionary relating RNA secondary and tertiary structure elements derived from experimentally determined RNA structures that often include non-canonical and pseudoknot interactions.

#### RMSD-based pairwise comparison and clustering of RNA tertiary structures

The global, pairwise comparison of RNA 3D structures was conducted using a root-mean-square deviation (RMSD) measure (Kabsch 1976). All pairs of RNA 3D models were superimposed and the RMSD scores were calculated using PyMOL 2.4 (Delano 2002).

Next, based on the RMSD matrix obtained from PyMOL 2.4, RNA 3D structures were clustered by employing OC cluster analysis program with default settings (single linkage algorithm) (Barton 2004). OC cluster analysis program was also used to calculate the centroids of the RNA 3D structure ensembles.

### In vitro validation

**Fig. 2:**
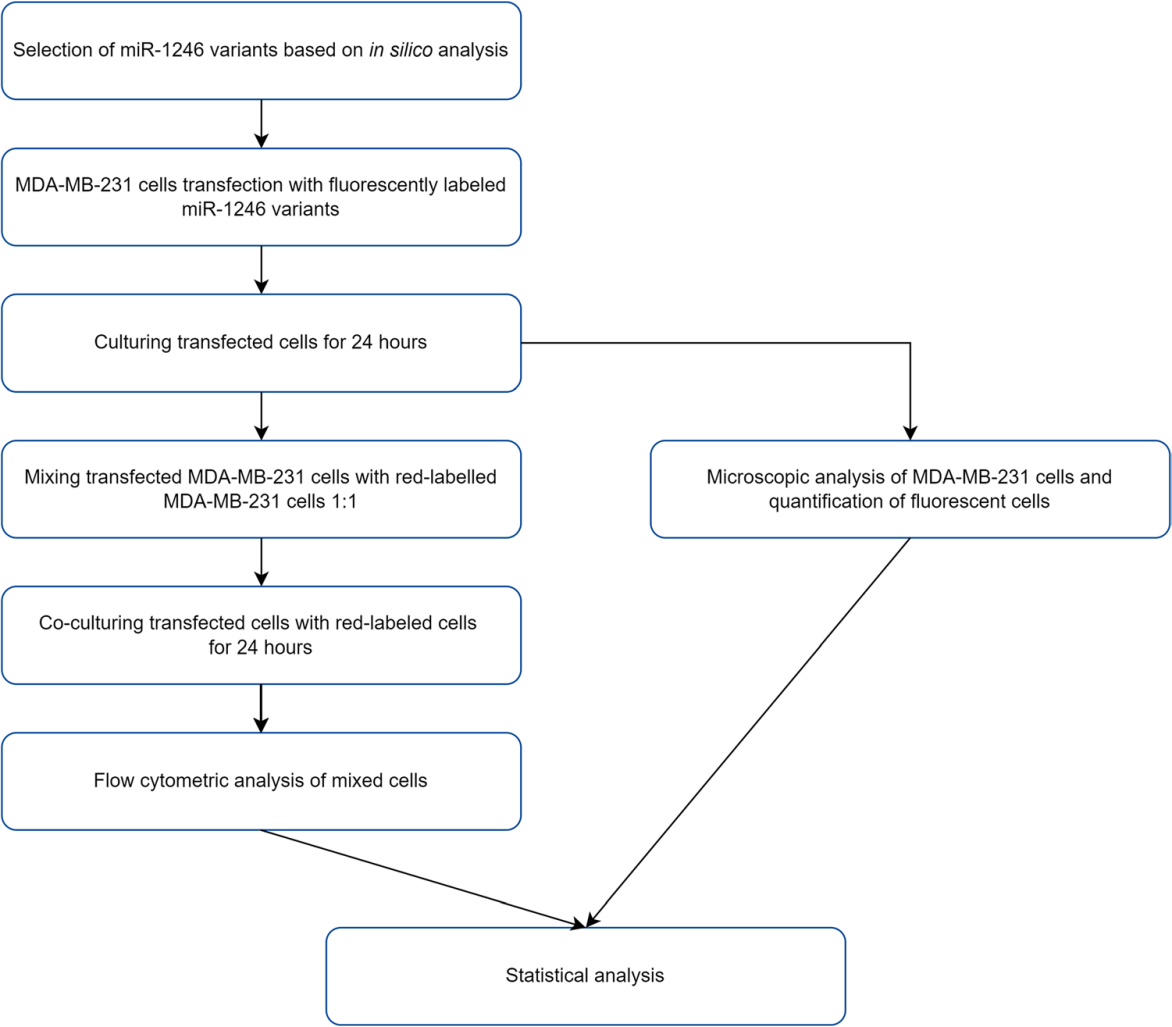
The workflow of the in vitro study

The subsequent steps of the in vitro analysis are presented in Fig. 2.

#### Generation of RNA probes

Based on the results of the in silico data analysis, we have prepared 5 double-stranded RNA probes numbered from 5 to 9. They differed from native miR-1246 by a single nucleotide within EXO-motif GGAG. Additionally, we have generated two random miRNA sequences of length equal to miR-1246, one containing only GGAG EXO-motif and the second only GCAG EXO-motif, where the location of those EXO-motifs was preserved (identical as in native miRNA), to show its importance in the control of RNA species loading into exosomes and extracellular transfer (c.f. Table 2). Random sequences were generated in such a way as not to contain any other EXO-motifs than GGAG or GCAG (Wei et al. 2021). Given that those control random miRNA (probe numbered 13 and 14 c.f. Table 2) shared with native miR-1246 only the sequence length and one of the EXO-motif, they were obviously not taken into account within in silico analysis.

We showed that a random sequence of length equal to miR-1246 and equipped only with GGAG (probe numbered 13, c.f. Table 4) or GGAC (probe numbered 14 c.f. Table 2) EXO-motif at the same location as in miR-1246 can be loaded and transferred from cell to cell.

**Table 2:**
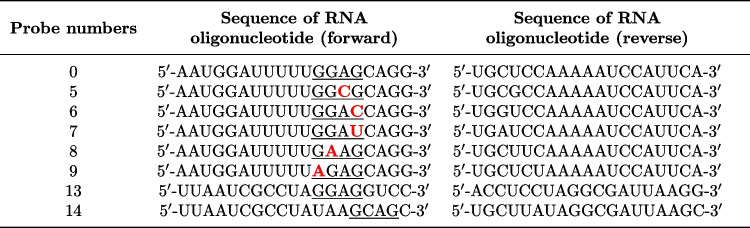
The sequences of RNA oligonucleotides used to obtain double-stranded RNA probes

Probe number 0 refers to the native miR-1246 mimic, while probes numbered 13 and 14 are random sequences with preserved EXO-motifs GGAG and GCAG respectively. Probes numbered from 5 to 9 differ from native miR-1246 by a single nucleotide within EXO-motif GGAG. The substituted nucleotides are denoted in red. Nucleotides forming EXO-motif are underlined. The probe numbers are consistent with miRNA sequence numbers in Table 2

#### Administration of probes to cells

Since the MDA-MB-231 cell line is a very well characterized and proven of secreting exosomes and expressing miR-1246 (Li et al. 2017a), it was selected for this study. It was obtained from the Department of Clinical Chemistry and Molecular Diagnostics of the K. Marcinkowski University of Medical Sciences in Poznań. MDA-MB-231/iRFP enriched with stable expression of the infrared fluorescent protein (iRFP) were obtained from the Polish Academy of Sciences, Institute of Human Genetics, Poznań, Poland. Cells were grown in RPMI1640 LO498 500 medium with glutamine (Biowest, Riverside, MO 64150, USA), with the addition of 10% fetal bovine serum (FBS, Biowest, cat number S181-100) and a 1% mixture of bactericidal and antifungal antibiotics (Antibiotic-Antimycotic, Biowest cat. 010). Cells were cultured in an incubator at 37$^{\circ }$C in a 5% CO$_{2}$ atmosphere in 75-cm$^{2}$ bottles (Googlab Scientific, Rokicin, Poland). In the next step, cells were digested with trypsin (Trypsin 10^®^, X0930, Biowest) — 5 min at 37$^{\circ }$C, followed by inactivation with complete medium and seeding of cells in 6-well culture plates (Thermofisher, Waltham, MA, USA) at 2.5 $\times$ 10$^{5}$ cells per well. Twenty-four hours after plating (90% confluency), double-stranded RNA probes were transfected (sequence of RNA oligonucleotides are listed in Table 2).

The probes were synthesized (Genomed, Warsaw, Poland) in series (c.f. Table 2) with modifications: phosphate groups at the 5$'$-end, five (probe nos. 0, 13, 14) and two (probe nos. 5–9) modified nucleotides with a methyl group (2$'$-OMe-RNA) and added fluorescent dye FITC at the 3$'$ end to one end of the two complementary oligonucleotides forming the duplex. 20 $\mu$M double-stranded RNA probes (duplexes) were prepared by mixing the appropriate 50 $\mu$M oligonucleotides in a buffer with a composition of 50 mM Tris, pH 8.0; 100 mM NaCl at the ratio of 30 $\mu$L of each oligo RNA with 15 $\mu$L buffer. Annealing was performed in a thermocycler by denaturing the probes at 92$^{\circ }$C and gradual cooling them to room temperature over 40 min (Biorad, Hercules, CA, USA). Lipofectamine 3000 Transfection Reagent (Thermofisher) was used to transfect cells. A transfection reagent was prepared by mixing 125 $\mu$L of serum-free RPMI1640 LO498 500 medium with 7.5 $\mu$L of Lipofectamine 3000. Separately 3.75 $\mu$L siRNA (20 $\mu$M) was mixed in 125 $\mu$L of serum-free medium. Both solutions were combined and left at room temperature for 15 min. After this time, 250 $\mu$L of the mixture was applied to the well with MDA-MB-231 cells. MDA-MB-231iRFP cells were not transfected. After 24 h, 2.5 $\times$ 10$^{5}$ transfected MDA-MB-231 and 2.5 $\times$ 10$^{5}$ non-transfected MDA-MB-231 iRFP cells were mixed and cultured on a well of a 6-well plate. After another 24 h, the cells were transferred into test tubes and cytometric analysis was performed.

#### Analysis using flow cytometry with imaging

The experiments were carried out on a FlowSight imaging flow cytometer (Amnis Merck Millipore, Darmstadt, Germany) equipped with 3 lasers (405 nm, 488 nm, and 642 nm), 5 fluorescence channels (acquisition in the form of a multi-channel CCD camera), and a laser scattered light detector — side scatter (SSC). Cell characterization was performed based on the following parameters:

1. The bright field of view derived from digital image processing of cells in (channel 1 — Ch01):
  - Gradient RMS (discrimination of objects/cells located in the plane of focus of the lens),
  - Aspect ratio (ratio of the short to the long axis of the analyzed object/cell),
  - Area (area of object/cells measured by the number of pixels)
2. From the side scatter light detector (SSC) in the form of the intensity of the scattered light in channel 6 (channel 6 — Ch06)
3. Fluorescent:
  - Green fluorescence (channel 2 — Ch02) from the FITC fluorochrome — acquisition in the form of the Intensity parameter,
  - Infrared fluorescence (channel 11 — Ch11) from iRFP fluorescent protein — acquisition in the form of the Intensity parameter
  - Area (area of object/cells measured by the number of pixels)

For the excitation of FITC and iRFP, a blue laser 488 nm and red laser 642 nm were used, respectively. Acquisition of cytometric data was performed based on specific laser settings and conversion of the collected fluorescence signals to a logarithmic scale. Data were analyzed using Ideas software (Amnis Merck Millipore). On a two-dimensional dot plot showing the intensity values (expressed in the so-called relative fluorescence units (RFU)) of green and red fluorescence (FITC vs iRFP), 3 types of cell sub-populations were defined: green fluorescence (FITC+), red fluorescence (iRFP+), and both types of fluorescence (FITC+/iRFP+). Each sample was analyzed in triplicate.

The interpretation of the results of the experiment consisted of marking:

- Percentage share of defined sub-populations (assessment of their distribution within the entire population of cells),
- Median values of the fluorescence intensity presented as the so-called relative fluorescence units — RFU (relative fluorescence units)

The fundamental change compared to the classical instrumental methods used in cell analysis is the fact that in flow cytometry with imaging, a significant part of the measured parameters are derivatives of digital image processing of cells in a bright field of view. The data obtained in this way refer to:

- Quality of obtained images of objects/cells (RMS gradient, contrast),
- The shape of the analyzed objects/cells (aspect ratio, elongatedness, shape ratio, circularity),
- The size of the analyzed objects/cells (area, diameter, height, length, width, spot area)

This information is correlated with the values of fluorescence intensity in individual channels read as averages for the entire object/cell or maximum/minimum values for individual pixels that make up the image of the object/cell.

#### Analysis of microscopic image

Additionally, 8.6 $\times$ 10$^{3}$ red MDA-MB-231iRFP cells were cultured in a 96-well plate to examine the differences in fluorescence of the nine probes. After 24 h, 100 $\mu$L of RNA probes was applied to the cells in the same way as in the main experiment, in triplicate for each probe. After 24 h, image analysis was performed using the ImageJ software (open-source scientific image processing and analysis software, NIH, USA). The experiment was performed in two independent replications.

#### Statistical analysis

Data was compiled using GraphPad Prism version 9.0.2 (GraphPad Software, USA). The significance level was set at 5% (*p* < 0.05). Cytometric and microscopic analyses were statistically calculated using one-way analysis of variance (ANOVA) with Tukey-Kramer multiple comparisons post-test. One-way analysis of variance (ANOVA) was performed using GraphPad InStat version 3.00 for Windows 95, GraphPad Software, San Diego, CA, USA (www.graphpad.com).

## Results

### In silico data analysis

#### Protein interactions prediction

First, we searched for the consensus motifs for RNA binding proteins (RBPs) in the analyzed miRNA sequences. As was expected, the native miR-1246 sequence contained the largest number of putative protein binding sites (c.f. Table 3). For miRNA sequences that differed from native one by a single nucleotide within EXO-motif GGAG, the number of motifs fell within the wide range of 2 to 16 (c.f. Table 3), with the highest number for the ones with numbers 1, 9, and 10 and the lowest for 6, 11, and 12. It is worth mentioning that the single substitution within the miRNA sequence has a great influence on the number of binding sites for RBPs.

**Table 3:** The list of RNA (miRNA) molecules used in the in silico data analysis ordered by the number of putative protein binding sites

RNA molecules (miRNAs) numbers	No. of potential consensus motifs for RBPs	No. of binding RBPs associated with consensus motifs	Ensemble Gene IDs
0	25	15	ENSG00000084072, ENSG00000092199, ENSG00000179172,
			ENSG00000066044, ENSG00000162374, ENSG00000136450,
			ENSG00000100650, ENSG00000111786, ENSG00000116001,
			ENSG00000161547, ENSG00000063244, ENSG00000100883,
			ENSG00000138385, ENSG00000153037, ENSG00000167881
1	16	12	ENSG00000084072, ENSG00000092199, ENSG00000125970,
			ENSG00000179172, ENSG00000066044, ENSG00000121774,
			ENSG00000116001, ENSG00000113742, ENSG00000011304,
			ENSG00000089127, ENSG00000141543, ENSG00000157349
9	14	13	ENSG00000084072, ENSG00000088247, ENSG00000169564,
			ENSG00000092199, ENSG00000107105, ENSG00000121774,
			ENSG00000135486, ENSG00000136450, ENSG00000138668,
			ENSG00000151923, ENSG00000161547, ENSG00000197111,
			ENSG00000214575
10	11	6	ENSG00000088247, ENSG00000096746, ENSG00000126945,
			ENSG00000169045, ENSG00000169813, ENSG00000132463
3	5	4	ENSG00000092199, ENSG00000107105, ENSG00000116001,
			ENSG00000135870
2	4	3	ENSG00000136450, ENSG00000111786, ENSG00000083896
7	3	3	ENSG00000139910, ENSG00000112081, ENSG00000065978
8	3	3	ENSG00000136450, ENSG00000111786, ENSG00000180210
5	3	3	ENSG00000107201, ENSG00000135829, ENSG00000160710
4	3	2	ENSG00000116001, ENSG00000167005
6	2	2	ENSG00000139910, ENSG00000111786
11	2	2	ENSG00000100650, ENSG00000104824
12	2	1	ENSG00000100650

RNA number 0 refers to the native miR-1246 sequence. RNAs numbered from 1 to 12 differ from native miR-1246 by single nucleotide within EXO-motif GGAG (c.f. Table 1). The last column contains the Ensemble Gene IDs list of putative proteins

#### RNA 2D structure modeling

**Fig. 3:**
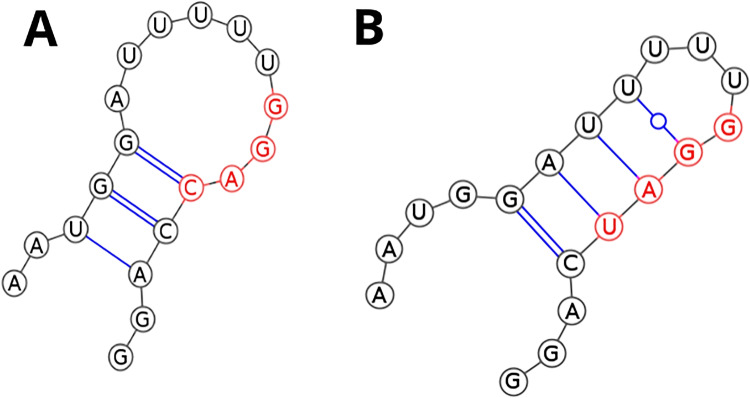
The 2D structures of miRNA 6 and 7, predicted by CentroidFold, CONTRAfold, and IPknot. **A** 2D structure of miRNA number 6. **B** 2D structure of miRNA number 7. Nucleotides forming EXO-motif are denoted in red

Next, RNA secondary structure modeling of all considered miRNA sequences was performed using CentroidFold (Sato et al. 2009), CONTRAfold (Do et al. 2006), and IPknot (Sato et al. 2011) which yielded consistent results. RNA molecules with numbers 0–5 and 8–12 did not form secondary structures while those numbered 6 and 7 both folded into hairpins (c.f. Fig. 3).

As it can be seen in Fig. 3, modified EXO-motif in miRNA with the number 6 is located within the loop of the hairpin while for miRNA numbered as 7, it is situated in the stem of the hairpin. Moreover, the hairpin in Fig. 3A has higher folding energy (−0.84 kcal/mol) than the one in Fig. 3B (−1.37 kcal/mol) and its loop is composed of 9 nucleotides contrary to hairpin in Fig. 3B which consists of 4 nucleotides. In general, miRNA with the number 7 should be more stable than the miRNA numbered 6.

It is worth mentioning that an experimental study has previously shown that many human miRNAs can form stable hairpins and that is facilitated by a cellular environment. The miRNA secondary structure affects both miRNA stability and function. The finding indicates that miRNAs and other short RNA hairpins can directly modulate protein activity (Belter et al. 2014).

#### RNA secondary structure clustering

In the next step, a pairwise comparison of all considered secondary structures was performed. As a result, two clusters were obtained, one containing 2D structures of miRNA with numbers 6 and 7 and another one composed of all other miRNA 2D structures (miRNA sequences with numbers 0–5 and 8–12).

#### RNA 3D structure modeling

Computational modeling of 3D structures of analyzed miRNA molecules was performed using RNAComposer. For each miRNA, up to 10 models were obtained for each secondary structure topology. It is worth mentioning that irrespective of the choice of the secondary structure prediction method, RNAComposer generated consistent and identical the resulting ensembles of 3D structures.

**Fig. 4:**
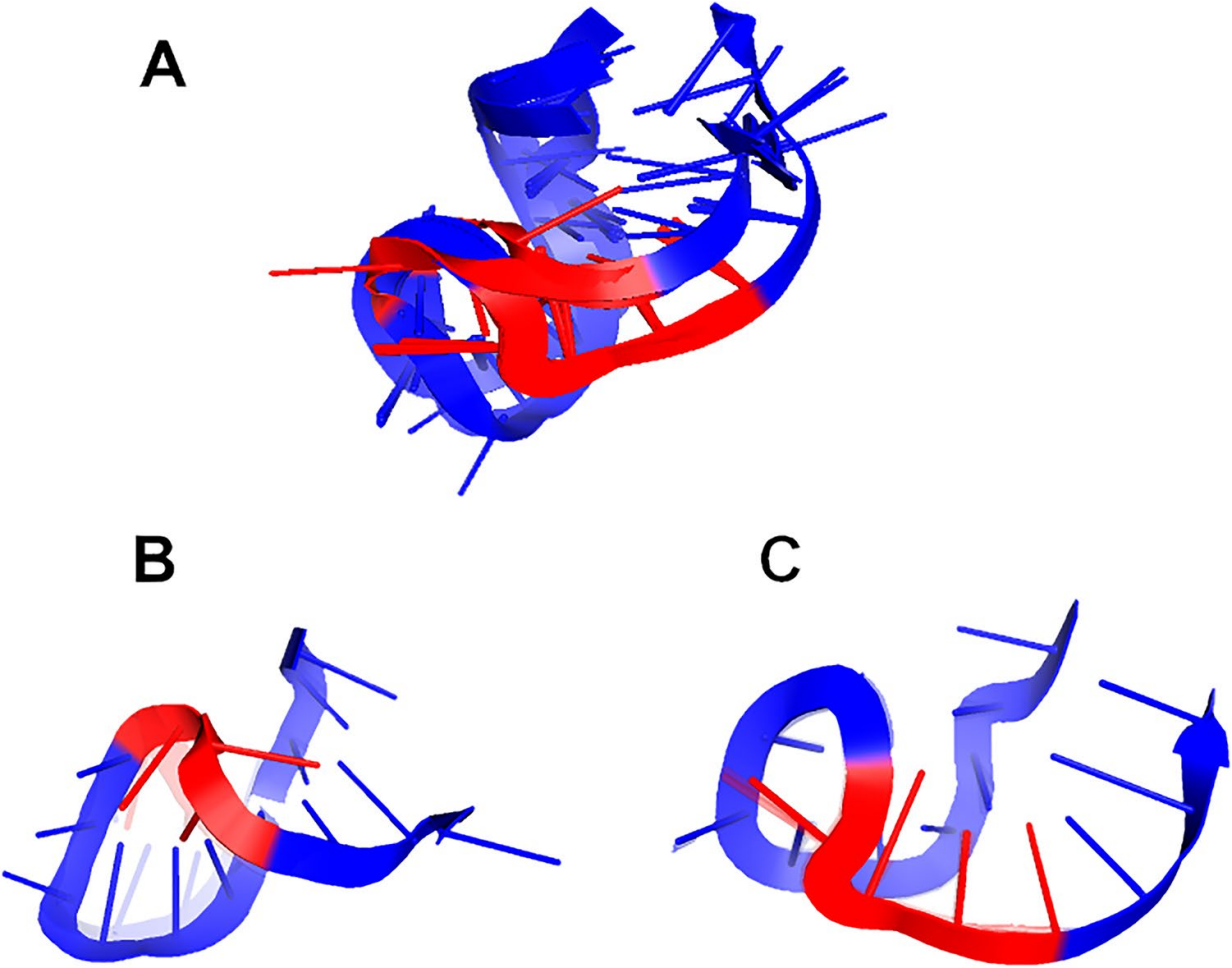
Visualization of the results of superposition of 3D structures of all analyzed miRNA molecules obtained using RNAComposer (identical for each RNAComposer-integrated algorithm for RNA 2D structure prediction: CentroidFold, CONTRAfold, and IPknot). Nucleotides forming analyzed EXO-motif are denoted in red. **A** The superposition of the whole ensemble of 3D structures was obtained for all analyzed miRNA. **B** The cluster containing miRNAs numbered as 6 and 7 (6 is the centroid of the whole cluster). **C** The cluster containing miRNAs numbered as 0, 1, 2, 3, 4, 5, 8, 9, 10, 11, and 12 (3 is the centroid of the whole cluster). For **B** and **C**, the centroid of the cluster is depicted in solid colors while the remaining cluster members are shown as transparent structures

In order to observe some preliminary differences in the global 3D folds of analyzed miRNA molecules, especially within the GGAG EXO-motif, we superimposed all predicted 3D structures using PyMOL 2.4 (c.f. Fig. 4A). As it can be seen in Fig. 4A, some 3D RNA models are quite diverse when it comes to the GGAG EXO-motif region.

#### RMSD-based pairwise comparison and clustering of RNA tertiary structures

First, the global pairwise comparison of 3D models within each ensemble (obtained for each miRNA sequence) was performed. For each ensemble of 3D structures, the values of extreme and average RMSD were calculated, and the centroid of the most populated cluster with an average distance to it was determined (c.f. Table 4). The aim was to select the best representative (the top-scoring ensemble member) for each ensemble.

**Table 4:** The results of RMSD-based analysis and comparison of RNA 3D models for each ensemble

RNA molecules (miRNAs) numbers	No. of 3D RNA models	Average RMSD (standard deviation)	RMSD min. max.	Average RMSD to centroid (standard deviation)
0	2	2.3 (0)	2.3 2.3	2.3 (0)
1	2	2.28 (0)	2.28 2.28	2.28 (0)
2	2	2.31 (0)	2.31 2.31	2.31 (0)
3	2	2.27 (0)	2.27 2.27	2.27 (0)
4	2	2.23 (0)	2.23 2.23	2.23 (0)
5	2	2.2 (0)	2.2 2.2	2.2 (0)
6	10	2.5 (0.76)	1.19 3.21	2.18 (0.26)
7	10	2.05 (0.82)	0.29 3.95	1.78 (0.73)
8	2	2.31 (0)	2.31 2.31	2.31 (0)
9	2	2.27 (0)	2.27 2.27	2.27 (0)
10	2	2.36 (0)	2.36 2.36	2.36 (0)
11	2	2.33 (0)	2.33 2.33	2.33 (0)
12	2	2.29 (0)	2.29 2.29	2.29 (0)

Next, the global, pairwise comparison of the centroids of all ensembles of RNA 3D structures, obtained in the previous step, was calculated. For each cluster of such 3D RNA structures, the following values were calculated: extreme and average RMSD together with standard deviation and the centroid of the whole cluster with the average distance to it (c.f. Table 5).

**Table 5:** The results of RMSD-based analysis and comparison of RNA 3D models for each ensemble

Cluster number	No. of 3D RNA models (numbers of miRNAs constituting a cluster)	Average RMSD (standard deviation)	RMSD min. max.	Average RMSD to centroid (standard deviation)
1	2 (6,7)	2.41 (0)	2.41 2.41	2.41 (0)
2	11 (0, 1, 2, 3, 4, 5, 8, 9, 10, 11, 12)	1.69 (0.05)	0.06 0.30	0.14 (0.05)

As it can be seen in Table 5 and Fig. 4A and B, two clusters were obtained, one composed of miRNAs numbered 6 and 7 and the second one of miRNAs with numbers 0–5 and 8–12. Since native miR-1246 (number 0) is a member of the second cluster and the folds of all molecules belonging to it are very similar (c.f. Fig. 4C), it is expected that the transfer capability of those miRNAs to the neighboring cells should be disturbed as little as possible. On the other hand, miRNAs constituting cluster number 1 are quite alike as far as their global 3D RNA structure is concerned, but there is a noticeable local difference within the 3D structure of the region involving EXO-motif and its immediate neighborhood (c.f. Fig. 4B).

#### Selection of miRNA sequences for in vitro validation

Based on the in silico analysis results, we decided to select for the in vitro verification miRNAs numbered 6 and 7, since as opposed to native ones and other modified miRNAs, they form a hairpin and belong to a separate cluster when their 3D fold is involved. Additionally, the single substitution in their sequence disturbs both miR-1246 EXO-motifs: GGAG and GCAG. For miRNA with number 6, the following modified EXO-motifs are obtained: GGAC and CCAG while for miRNA numbered 7: GGAU and UCAG.

We chose also miRNAs with numbers 5, 8, and 9 for in vitro validation. Although their 2D and 3D structures are similar to native miR-1246, they differ in the number of binding sites for RBPs.

### In vitro validation

#### The results of the analysis using flow cytometry with imaging

**Fig. 5:**
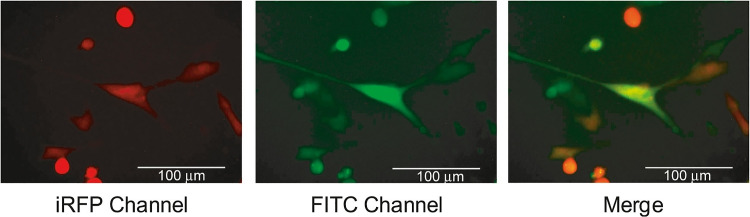
The image of MDA-MB-231 cells transfected with FITC-labeled double-stranded RNA probe no. 0. Probe no. 0 was introduced into the cells by transfection using Lipofectamine 3000. Twenty-four hours after transfection, the transfected cells were mixed 1:1 with MDA-MB-231 cells stably expressing the iRFP protein. Mixed populations were co-cultured for 24 h. Cells were analyzed with an EVOS (ThermoScientific) microscope and 40$\times$ objective. Two detection channels were used for iRFP (EVOS Cube No. 4656) and FITC (EVOS Cube No. 4651). The third panel shows the merged images obtained in both channels of the selected field containing doubly stained cells

**Fig. 6:**
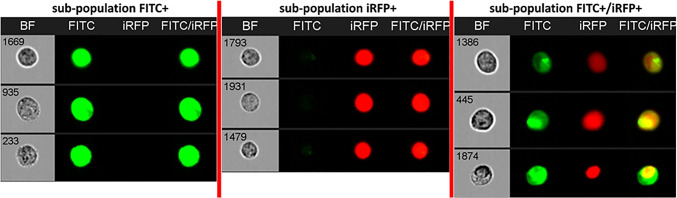
The examples of the images from sub-populations FITC+, iRFP+, and double-positive — FITC+/iRFP+ — analyzed using imaging flow cytometry (FlowSight Amnis Merck Millipore, Darmstadt, Germany). Channels presenting images are BF — brightfield, FITC — green fluorescence signals from FITC, iRFP — red fluorescence signals from iRFP, and FITC/iRFP — merged images of FITC and iRFP fluorescence intensity signals. Image of MDA-MB-231 cells transfected with FITC-labeled double-stranded RNA probe no. 0. Probe no. 0 was introduced into the cells by transfection using Lipofectamine 3000. Twenty-four hours after transfection, the transfected cells were mixed 1:1 with MDA-MB-231 cells stably expressing the iRFP protein. Mixed populations were co-cultured for 24 h

**Fig. 7:**
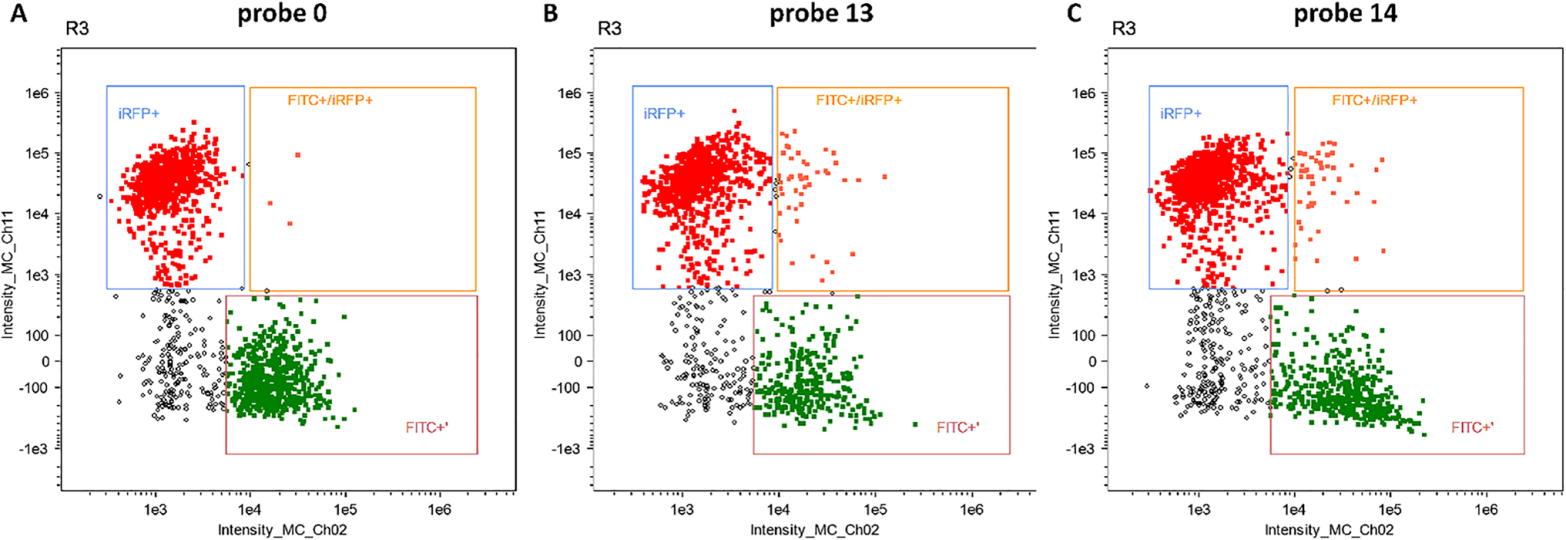
The results of cytometric analysis of MDA-MB-231 cells transfected with double-stranded RNA probes labeled with FITC dye. The probes numbered 0 (**A**), 13 (**B**), and 14 (**C**) were introduced into the cells by transfection using Lipofectamine 3000. Twenty-four hours after transfection, the transfected cells were mixed 1:1 with MDA-MB-231 cells stably expressing the iRFP protein. Mixed populations were re-cultured for 24 h. Cells were analyzed by flow cytometry. Two detection channels were used for iRFP (Intensity_MC_Ch11) and FITC (Intensity_MC_Ch02). Selected representative cytograms show the gating scheme, iRFP+, iRFP+/FITC+, and FITC+$'$

**Fig. 8:**
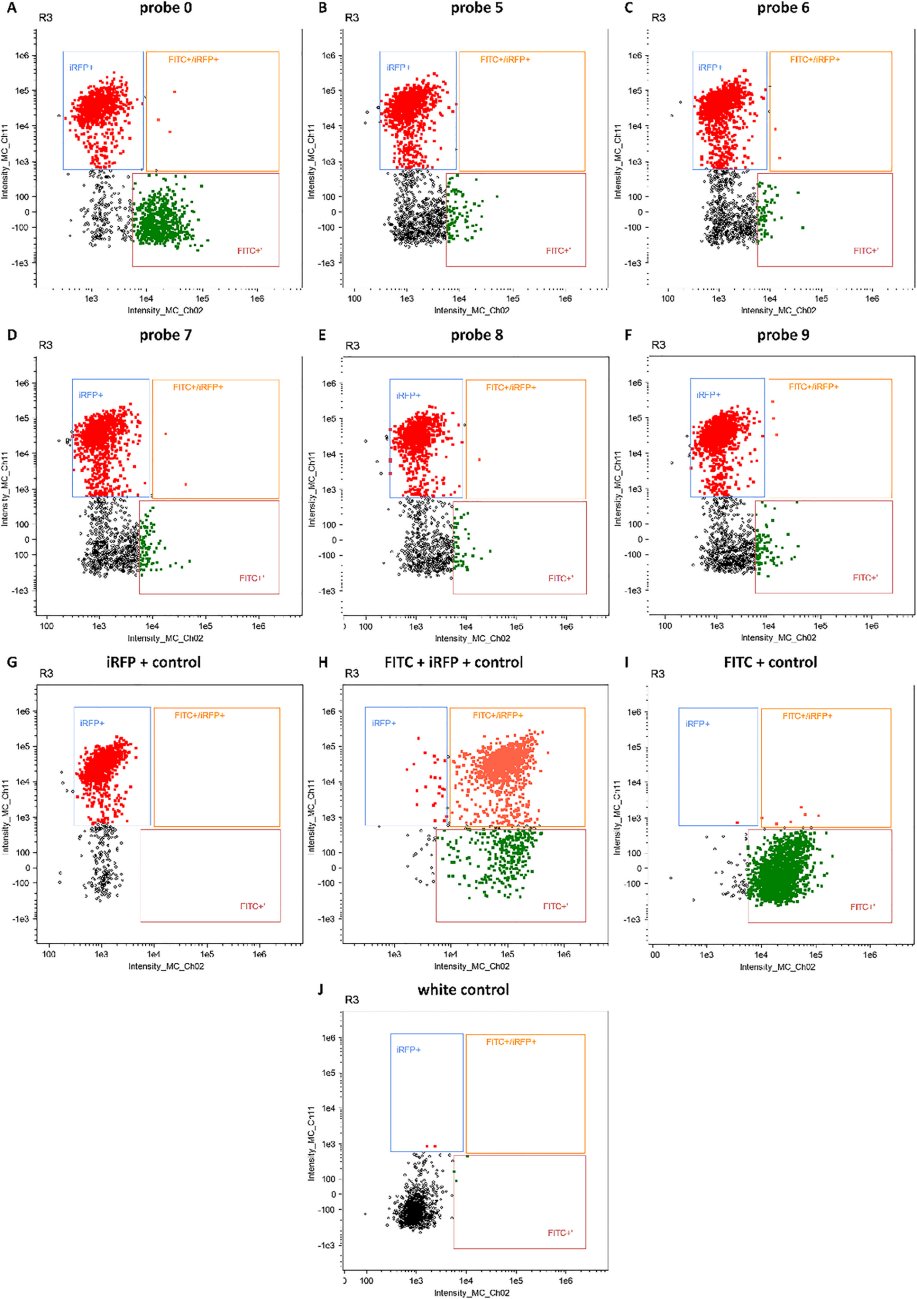
The results of cytometric analysis of MDA-MB-231 cells transfected with double-stranded RNA probes labeled with FITC dye. The probes numbered 0 and 5–9 (**A**–**F**) were introduced into the cells by transfection using Lipofectamine 3000. Twenty-four hours after transfection, the transfected cells were mixed 1:1 with MDA-MB-231 cells stably expressing the iRFP protein. Mixed populations were re-cultured for 24 h. Cells were analyzed by flow cytometry. Two detection channels were used for iRFP (Intensity_MC_Ch11) and FITC (Intensity_MC_Ch02). Selected representative cytograms show the gating scheme, iRFP+, iRFP+/FITC+, and FITC+$'$. Controls **G**–**J** — three gates were established by analysis of control cells expressing iRFP protein (iRFP+ gate) (**G**) or the iRFP cells transfected with the FITC dye probe (iRFP/FITC+$'$ gate) (**H**), or only the FITC dye probes (FITC+$'$ gate) (**I**) and native non-transfected MDA-MB-231 cells (**J**)

The breast cancer cell line MDA-MB-231 was used to test the oligonucleotide miRNA transfer to the neighboring cells. The probes were administered to the cells by transfection. Twenty-four hours after transfection, the cells were mixed 1:1 with MDA-MB-231 cells stably expressing the iRFP protein. Mixed populations were co-cultured for 24 h (c.f. Fig. 5). Co-cultured cells were analyzed using a flow cytometer (c.f. Figs. 6, 7, and 8). Two detection channels were used for FITC and iRFP, respectively. Examples of the single-cell images from sub-populations FITC+, iRFP+, and double-positive — FITC+/iRFP+ — were obtained using imaging flow cytometry (FlowSight Amnis Merck Millipore, Darmstadt, Germany) and are presented in Fig. 6. Cytograms are shown in Figs. 7 and 8.

**Fig. 9:**
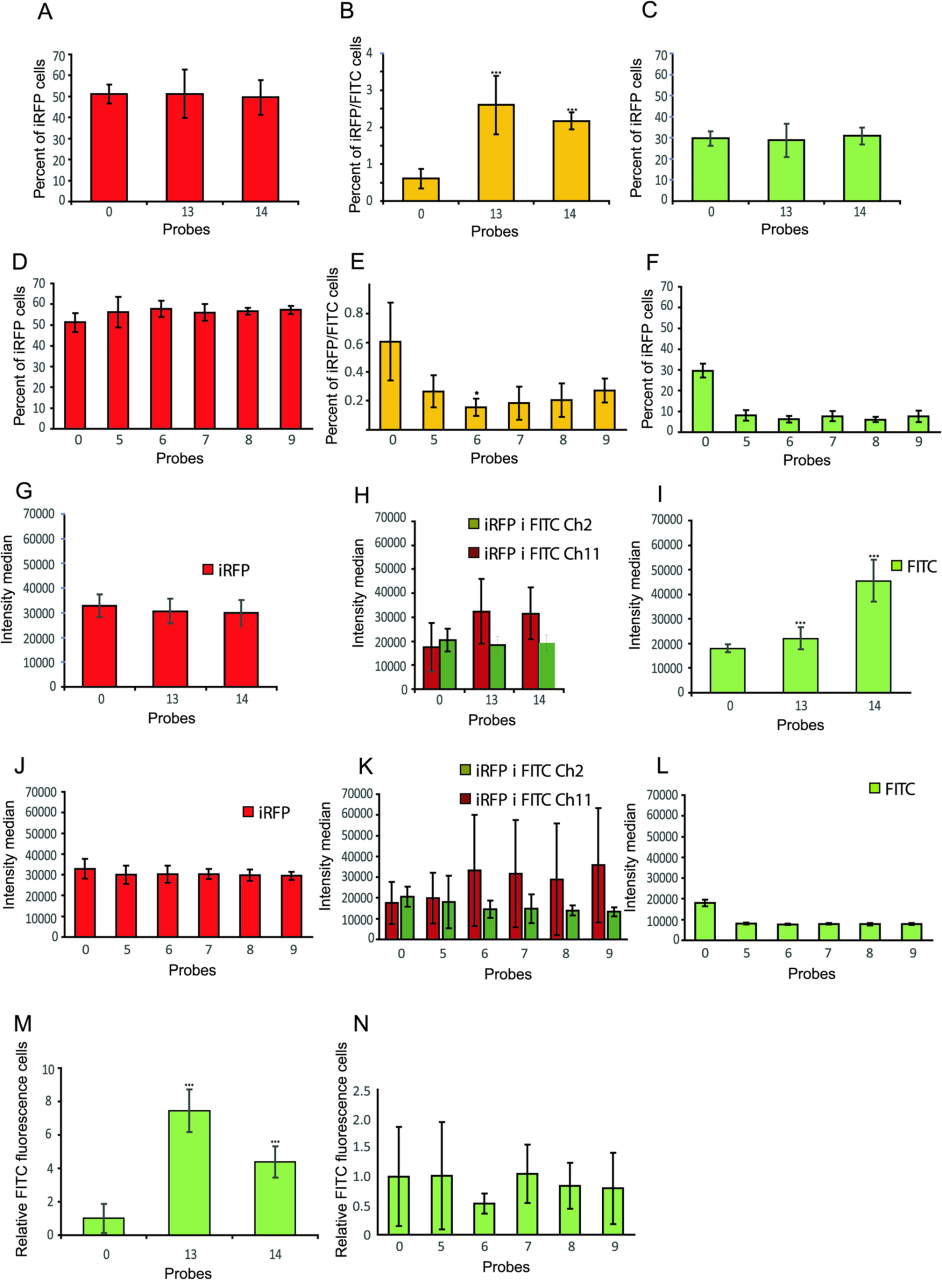
The quantitative presentation of results obtained by cytometric and microscopic analysis of MDA-MB-231 cells transfected with double-stranded RNA probes 0, 5, 6, 7, 8, 9, 13, and 14, labeled with FITC dye. The probes were introduced into the cells by transfection, using Lipofectamine 3000. **A**–**L** Twenty-four hours after transfection, the transfected cells were mixed 1:1 with MDA-MB-231 cells stably expressing the iRFP protein. Mixed populations were re-cultured for 24 h. Cells were analyzed by flow cytometry. Two detection channels were used for iRFP and FITC. The diagrams show the mean percentage of gated populations (**A**–**F**) and median intensity (**G**–**L**) corresponding to the iRFP+, iRFP+/FITC+$'$, and FITC+$'$ gates. Four biological replicates of the experiment were used and each cell sample was cytometrically measured 3 times for a total of 12 measurements. *p < 0.05, ***p < 0.001, whiskers are OD. **M**, **N** The transfected, non-mixed cells were also photographed using the microscopic camera, and obtained pictures were analyzed with ImageJ software. The graphs show the mean fluorescence intensity estimated by analyzing randomly photographed areas from at least three different cell culture wells ***p < 0.001, whiskers are OD

Upon the same cell samples, quantitative cytometric analysis was done. We calculated the average values of the percentage of gated cells and median fluorescence intensity. They are shown in Fig. 9A–C, D–F and G–I, J–L. Thus, in Fig. 9A–C and D–F, the *X*-axis plots the probe number and the *Y*-axis represents the percentage of fluorescent cells. Independently of applied miRNA duplex (probe nos. 0, 5–9 and 13, 14), MDA-MB-231iRFP cells showed very similar population percentages of about 50% in iRFP+ gate (Fig. 9A and D).

In Fig. 9B and E, we show the percentage of cells with double fluorescence corresponding to iRFP and FITC. The percentage of cells doubly stained is very low from 0.15 to 0.65 if comparing only probes numbered 0 and 5–9. The lowest value was observed for probe no. 6 and the highest for probe no. 0 (native miR-1246 probe containing GGAG EXO-motif). Unexpectedly, random probe with GGAG sequence (RNA duplex no. 13) represented 2.5% of doubly stained cells. We supposed that this mixed population was mainly MDA-MB-231iRFP cells, which incorporated the miRNA-FITC probes from transfected MDA-MB-231 cells. It was shown that the number of MDA-MB-231iRFP cells assimilating probe no. 6 was lower than the number of MDA-MB-231iRFP cells containing probe no. 5. This may suggest decreased transfer of probe no. 6 between cells or its decreased stability in cells or transfection efficiency.

These results are consistent with the above-described differences in properties of probe nos. 5 and 6 connected with the differences in sequence and structure (c.f. “In silico data analysis” section). In probe no. 5, the sixth base (A) from the 3$'$ end of the miR-1246 native sequence is replaced by C (native probe no. 0: 5$'$-GGAG-3$'$, probe no. 5: 5$'$-GGCG-3$'$), while in probe no. 6, the fifth base (G) from the 3$'$ end of the miR-1246 native sequence is replaced by C (native probe no. 0: 5$'$-GGAG-3$'$, probe no. 6: 5$'$-GGAC-3$'$). It is worth noticing that probe no. 7 was similarly to probe no. 6 transferred to the adjacent cells at a low level, but higher than for probe no. 6.

In Fig. 9C and F, MDA-MB-231 cells transfected with FITC probe nos. 5–9 accounted for about 6–8% of the analyzed cell population. Probe nos. 6 and 8 represented the lowest percentage of the population. MDA-MB-231 cells transfected with FITC probe nos. 0 and 13, accounted for about 30% of the total cell population.

In Fig. 9G–I and J–L, median fluorescence intensity is shown, reflecting an average fluorescence of probes in cells. The median iRFP fluorescence for all probes was the same (approx. 30,000 RFU relative fluorescence units) (c.f. Fig. 9G and J).

The median FITC fluorescence of probe nos. 5–9 was also very similar (approx. 8000 RFU) (c.f. Fig. 9L). Fluorescence of probe nos. 0, 1, and 14 was approx. 18,000–20,000 RFU (c.f. Fig. 9I). We analyzed the median fluorescence of a small number of cells from the doubly stained population in channel 2 (FITC) and channel 11 (iRFP). This population performed approx. 15,000–18,000 RFU in channel 2 and 30,000 RFU in channel 11.

Probe nos. 0, 13, and 14 showed higher fluorescence in cells than probe nos. 5–9 (Fig. 9H and L), although all of them had comparable fluorescence before being transfected into the cells. The probes numbered 0, 13, and 14 differ from the probes of series nos. 5–9 in the number of methylated ribose molecules, 20 riboses (10 on each strand) and four riboses (two on each strand), respectively. Presumably, this difference may have affected the stability of RNA duplexes in the cells.

Concluding, we observed a lack of differences in the median fluorescence (FITC+$'$ gate) of cells transfected by probe nos. 5–9, measured by flow cytometry. Despite this, in the cells from the mixed population, in the iRFP/FITC+$'$ (gate iRFP and FITC), a quantitative difference was observed between the percentage of cells transfected with probe nos. 5 and 6 (c.f. Fig. 9D–F), which may suggest the impact of probe sequence and structure on the transfer efficiency between cells.

#### Analysis of microscopic image

MDA-MB-231 cells transfected with the probes (before mixing with MDA-MB-231 cells stably expressing the iRFP protein) were also analyzed using a fluorescence microscope and ImageJ software. With their help, the fluorescence level in cells was determined 24 h after transfection.

As it can be seen in Fig. 9M, for probe nos. 13 and 14, the fluorescence is higher as compared to probe no. 0 (c.f. Fig. 9B). It may suggest that the difference in fluorescence level in cells after mixing transfected cells with MDA-MB-231 cells stably expressing the iRFP protein is caused by the stability of RNA duplexes and emerges before the cells are mixed.

On the other hand, the cells containing probe nos. 5–9 showed similar fluorescence in the microscopic image 24 h after transfection regardless of the probe used (c.f. Fig. 9N). This result is consistent with the measurement of cell fluorescence with a cytometer (c.f. Fig. 9F). It may further suggest the influence of the miRNA sequence and structure on the efficiency of its intercellular transfer.

#### Results summary

The obtained results indicate the importance of probe sequences and structures in their stability in cells and intercellular transport.

## Discussion

Exosomes (extracellular vesicles, EVs) are natural, intracellular carrier systems that are known to contain biomolecules such as proteins, mRNAs, and miRNAs. Recently, an increasing number of studies have focused on EVs due to their involvement in tumor cells communication resulting in tumor growth and progression (Yang and Robbins 2011; Torii et al. 2021) and due to newly discovered mechanisms through which their miRNA cargo is controlled (Villarroya-Beltri et al. 2013; Zhang et al. 2015; Groot and Lee 2020). RNA binding proteins (RBPs) such as hnRNPA2B1 have been proved to be involved in selective miRNA sorting into EVs through recognizing specific motifs in RNA (EXO-motifs). However, these sequence-dependent mechanisms are still largely unknown (Groot and Lee 2020).

One of the exosomal miRNAs that have recently attracted the attention of many researchers is miR-1246, which was first recognized by an application of a high throughput sequencing technique in human embryonic stem cells (Xie et al. 2019; Ghafouri-Fard et al. 2022). It has been demonstrated that miR-1246 acts as a proto-oncogene in many cancers such as lung and breast cancer and it was also identified in highly metastatic tumor EVs, where this miRNA was shown to have a role in acquiring drug resistance by tumor endothelial cells. Unfortunately, the detailed mechanisms by which highly metastatic tumor-secreted exosomes become enriched with miR-1246 remain unclear (Dai et al. 2021; Torii et al. 2021).

MiR-1246 and miR-122-5p (Li et al. 2021) contain two potential EXO-motifs: GGAG and GCAG, of which only GCAG has previously been analyzed (Xu et al. 2019). However, to our knowledge, none of those EXO-motifs has been studied as an important factor regulating the miR-1246 stability and intercellular transfer. Since GGAG is one of the most typical EXO-motifs present in many exosomal miRNAs, including miR-1246 (Villarroya-Beltri et al. 2013; Wei et al. 2021), and is bound by hnRNPA2B1 protein, which was i.e. shown to selectively guide miRNA-155-5p packaging into lung cancer EVs (Ingenito et al. 2019; Li et al. 2021), we decided to explore whether it can also mediate miR-1246 transfer into exosomes. Our research aimed to check whether single substitutions within GGAG EXO-motif can influence the intercellular transfer of miR-1246.

First, to estimate the impact of single nucleotide substitutions on miR-1246 intracellular transfer and stability, an in silico analysis of all miR-1246 variants that differed from native one by single substitution within EXO-motif GGAG was conducted. In its first step, we performed computational research to find potential proteins interacting with miR-1246 variants using our approach based on the ATtRACT database of RBPs consensus motifs and homologues genes of RBPs. As a result, we could observe that the number of RPBs binding sites for analyzed miRNAs fell within the very wide range of 2 to 25 (c.f. Table 3), with the highest number for native miR-1246 sequence. It is worth mentioning that the single substitution in GGAG EXO-motif within miR-1246 greatly impacts the number of binding sites for RBPs. However, it should be also noted that substrate selection by a given protein is rarely binary and that RBPs tolerate often some degree of sequence variation within binding sites (Mitchell and Parker 2014; Jankowsky and Harris 2015). Moreover, every interaction between a given protein and the RNA’s binding site depends on the RNA and protein concentrations, competition among other proteins for RNA, and binding context (Jankowsky and Harris 2015; Kazan et al. 2010). In particular, the secondary and tertiary structure of RNA can influence the binding affinity of some RBPs (Corley et al. 2020; Gronning et al. 2020).

Therefore, in the next step, we examined the secondary structures of all miR-1246 variants together with the native miR-1246 sequence, applying three commonly used 2D prediction programs, namely CentroidFold, CONTRAfold, and IPknot (Popenda et al. 2012; Antczak et al. 2019). We used the DBSCAN method to find similarities between all 2D structures and obtained two clusters. It turned out that two of the analyzed miRNA sequences (constituting one cluster) adopted a hairpin structure while the remaining ones stayed single-stranded (c.f. Fig. 3). Those two miRNA molecules that formed hairpin structures had the following single nucleotide modifications within EXO-motif GGAG: G to C substitution at position 15 of miR-1246 sequence (miRNA numbered 6 with modified EXO-motif GGAC) and G to U substitution at position 15 of miR-1246 sequence (miRNA numbered 7 with modified EXO-motif GGAU). It is interesting to note that the location of the modified EXO-motifs within hairpins was different (c.f. Fig. 3), namely GGAC of miRNA numbered 6 was situated within a loop of the hairpin while GGAU for miRNA numbered as 7, in the stem of the hairpin. Additionally, taking into account the folding energy ${\Delta }$G of both miRNAs, miRNA with the number 7 should be more stable than the miRNA numbered 6. Those observations are consistent with the existing literature, where it has been shown that miRNAs stability correlates with ${\Delta }$G of their structure and miRNA 2D structure affects both miRNA stability and function (Belter et al. 2014).

In the last step of the in silico analysis, we performed the computational modeling of 3D structures of analyzed miRNA molecules using RNAComposer and we conducted clustering to investigate the similarities among them. Similarly, as in 2D structure clustering, we obtained two ensembles of structures. miRNAs numbered 6 and 7 belonged to the same cluster and we could observe a noticeable local difference in their 3D structures within the region involving modified GGAG EXO-motif and its immediate neighborhood (c.f. Fig. 4B). Moreover, the single substitution within miRNA numbered 6 and 7 sequence disturbs both EXO-motifs present in miR-1246: GGAG and GCAG. Hence, we decided to primarily select them for in vitro verification. Taking into account the difference in the number of binding sites for RBPs, we chose also for in vitro validation miRNAs with numbers 5 and 8 having 3 RBPs binding sites and 9 holding 14 RBPs consensus motifs (c.f. Table 3).

The influence of mutations within potential EXO-motifs on selective packaging into EVs has been previously explored in Temoche-Diaz et al. (2019). Here, two new La-binding EXO-motifs embedded in the miR-122 sequence were detected, using biochemical and genetic tools. However, the authors did not analyze the impact of single substitutions within EXO-motifs, but the effect of the whole motif replacement. Contrary to our approach, they did not consider the secondary and tertiary structure of miRNA and its variants.

As a result of investigations of RNA sorting into EVs, it has been postulated that recognizing secondary rather than primary RNA sequence motifs should be taken into account (Sadik et al. 2018). This is reflected in the research reported in Shurtleff et al. (2016), where the RNA-binding protein Y-box I (YBX-1) was proposed to be involved in escorting miRNAs and other exosomal RNAs into exosomes. Since it is known that YBX1 recognizes and binds hairpin-loops in a murine retrovirus, it enhances the probability that the recognition motif for sorting into exosomes may be based on RNA secondary and/or tertiary structure rather than RNA sequence (Bann et al. 2014).

Next, in the in vitro validation part of our approach, selected miR-1246 sequences having single substitutions within GGAG EXO-motif were introduced as miRNA FITC labeled at 3$'$ end duplexes to breast cancer cell line MDA-MB-231. Since it remains a critical issue that exogenous miRNA mimics introduced directly into cell cytosol seem to be unstable and are at risk of rapid degradation by nucleases, also their efficiency to cross cell membranes is low (Zhang et al. 2012; Nogimori et al. 2019), we introduced them as chemically modified oligonucleotides. It has been shown that synthetic miRNAs containing 2′-fluoro, 2′-O-methyl, or 2′-O-methoxyethyl modified nucleotides could regulate gene expression by Ago2-dependent mechanism (Matsui et al. 2016). Moreover, such an approach significantly increases the resistance of miRNA mimics to nuclease degradation, while at the same time ensuring that the interaction of such molecules with target mRNA and RBPs remains unaffected (Hyjek-Skladanowska et al. 2020).

After 24 h, the transfected cells were mixed with MDA-MB-231 cells stably expressing the iRFP (infrared fluorescent protein) protein. Next, mixed populations were grown and co-cultured for 24 h. The co-cultured cells were analyzed by flow cytometry and fluorescent microscopy. It is worth mentioning that flow cytometric analysis is commonly used to study the intercellular exchange of miRNA (Aucher et al. 2013).

As a result, the highest quantitative difference in transfer efficiency could be observed between probe nos. 5 and 6, with the lowest value for probe no. 6 (c.f. Fig. 9D–F). Probe numbered 7 was also the one that was sorted to the neighboring cells at a low level, but higher than probe no. 6. There was no significant difference between probe nos. 5, 8, and 9 as far as the level of transfer was concerned (c.f. Fig. 9D–F). We showed also that a random sequence of length equal to miR-1246 and equipped only with GGAG or GGAC EXO-motif at the same location as in native miR-1246 can be transferred through EVs (c.f. Fig. 9A–C, G–I).

It shows that single nucleotide substitutions within miR-1246 GGAG EXO-motif which leave the second GCAG EXO-motif intact do not influence significantly the sorting of miR-1246 into intercellular transfer. On the other hand, the single nucleotide substitutions that disturbed both motifs (last position of GGAG and first of GCAG) slightly decreased the level of miR-1246 in intracellular transfer. Probably the primary (sequence), secondary, and tertiary structure of those miRNAs modulated the binding affinity of RBPs responsible for loading into exosomes. Since they tolerate some sequence variations, as a result, the transfer was altered but not completely stopped.

Moreover, single nucleotide modifications within miRNA sequence can influence miRNA stability. It has been postulated that at least two mechanisms regulate miRNA stability apart from terminal methylation, and are dependent on miRNA sequence. These are the AGO protein binding and target sequence-dependent mechanisms (Zhang et al. 2022). RBPs such as Ago2 are common factors responsible for miRNA stability in mammalian cells because they protect miRNAs from degradation by RNases. Moreover, it has recently been shown that total miRNA is in excess of Ago proteins so miRNA can exist in the cell as a free molecule, not knotted in the RISC complex (Janas et al. 2012; Belter et al. 2014). Therefore, the differences in secondary and tertiary structure within miRNAs may result in a distinct turnover time for them.

Target mRNA recognition by miRNA in complex with AGO occurs primarily through the seed region located on the 5$'$-end of miRNA (Sheu-Gruttadauria et al. 2019). 3′-end nucleotides beyond the seed are also important for target interactions. Therefore, modification of the 3$'$ end GGAG motif could influence the stability of miR-1246 variants in our study. Such extended complementarity, beyond seed sequence, was observed for *CCNG2* 3$'$UTR, in which the GGAG motif is complementary to the CCUC sequence in the target gene (Li et al. 2017b).

Our results can also suggest that both GGAG and GCAG EXO-motifs can influence miR-1246 intercellular transfer in an independent manner and additionally, some EXO-motif alterations can modulate intracellular shearing of miR-1246.

## Conclusions

The present study constitutes a novel insight into the role of single nucleotide substitutions within miR-1246 and their influence on miR-1246 stability and intracellular transfer. Through the in silico and in vitro analysis, we showed that GGAG and GCAG motifs probably can regulate miR-1246 sorting and stability, in a state of independence from one another. Moreover, single nucleotide substitutions that disturbed both motifs were able to influence the level of miRNA-1246 transferred to the neighboring cells.

In this work, we used in silico and in vitro methods as a complementary approaches. In this way, we demonstrated how computer modeling can narrow the spectra of molecules, which are worth synthesizing. Thus, it constitutes a valuable and practical resource to biologists, since such an approach reduces the cost of wet lab experiments.

Such an approach could be applied to search for new miRNA candidates as potential markers and to improve designing the new therapeutics. However, further studies are required to adjust computer modeling to increase the predictability of miRNA stability and transfer in living cells.
